# Subxiphoid vs transthoracic approach thoracoscopic surgery for spontaneous pneumothorax: a propensity score-matched analysis

**DOI:** 10.1186/s12893-019-0503-y

**Published:** 2019-04-29

**Authors:** Liang Chen, Feng Liu, Bin Wang, Keping Wang

**Affiliations:** Department of Thoracic Surgery, Nanjing Chest Hospital, 215 Guangzhou Road, Nanjing City, Jiangsu Province, China

**Keywords:** Subxiphoid, Thoracoscopic, Spontaneous pneumothorax

## Abstract

**Background:**

The transthoracic thoracoscopic surgery is currently accepted as a favorable technique for bullectomy for primary spontaneous pneumothorax. Recently, uniportal subxiphoid thoracoscopic surgery has been proposed as an alternative to conventional transthoracic thoracoscopic surgery.

**Methods:**

From November 2014 and January 2016, 127 consecutive patients who met the inclusion criteria were enrolled in this study. Among these patients, 32 were treated using subxipoid approach, whereas 95 were treated using transthoracic approach. Propensity score case-matching was performed to adjust for patient backgrounds.

**Results:**

The two groups of 32 pairs were well matched for baseline and surgical characteristics. Patients who underwent subxipoid approach had a longer operation time than transthoracic approach (*p* = 0.004). The subgroup analysis showed that the operation time for bilateral bullectomy was similar between the groups (*p* = 0.986). There were no differences between the groups with respect to the hospital stay after surgery, chest drain duration, the number of the staples used for the operation, and postoperative recurrence. However, the provoked arrhythmias events during surgery were significantly higher in the subxiphoid approach group (*p* = 0.011).

**Conclusions:**

Although transthoracic thoracoscopic surgery for spontaneous pneumothorax is well established, uniportal subxiphoid thoracoscopic surgery may be a potentially alternative way to management of patients with spontaneous pneumothorax in selected cases, especially for bilateral surgery, but causions should be taked.

## Background

Spontaneous pneumothorax is defined as the presence of gas in the pleural space which is between the chest wall and the lung [1]. The surgical treatment could decrease the recurrence rate. Video-assisted thoracoscopic surgery improves fast-track recovery in the management of spontaneous pneumothorax [2].

Conventional thoracoscopic surgery is performed via transthoracic approach with surgical incisions from three to two, and then to single one [3–5]. Rencently, The subxiphoid thoracoscopic surgery has been reported as a novel technique, and even it has been used to major lung resections [6, 7].We herein describe our technique for performing uniportal subxiphoid thoracoscopic in patients with spontaneous pneumothorax and compare outcomes with transthoracic thoracoscopic surgery for spontaneous pneumothorax.

## Methods

Between November 2014 and January 2016, we retrospectively reviewed the surgical records of 127 consecutive patients with spontaneous pneumothorax who received thoracoscopic surgery at Nanjing chest hospital.

The eligibility criteria for this study include recurrent episode, persist air leakage after chest tube drainage for more than 3 days, or visible pulmonary bulla on computed tomography.

The exclusion criteria for this study include a history of lung diseases, such as chronic obstructive pulmonary disease, pulmonary tuberculosis and a history of ipsilateral thoracic surgery. The difference of these two methods, such as incisions, surgical risks, and potential complications were fully introduced to each patient before operation. The present study was approved by the ethics committee of our hospital.

### Propensity score matching

To minimise the influence of clinical confounders on outcomes, propensity score matching between the two groups was performed to create comparable groups of patients. A 1–1 ratio was used for the present propensity score matching. The propensity score was constructed by using clinical variables such as sex, age, side involved and body mass index (BMI). Finally, the propensity score yielded two well-matched 32 pairs each, including 32 who underwent subxiphoid approach, 32 who underwent transthoracic approach.

### Surgical technique

The subxiphoid approach made under general anesthesia using one-lung ventilation was performed with a 3 cm skin incision made over the subxiphoid area, and a substernal tunnel was created from the wound to thoracic cavity. The patient was placed in the horizontal decubitus position. A 10-mm 30°angled video thoracoscope along with surgical instruments are then placed through the incision.

The transthoracic approach was made under general anesthesia using one-lung ventilation, and the patient was placed in a lateral position.

A 10-mm 30-degree thoracoscope was mostly introduced through the seventh intercostal space in the mid-axillary line. The working port and accessory port were made at the fifth intercostal space in the anterior axillary and the sixth intercostal space in the posterior axillary line, respectively.

In all cases, the bullectomy was performed with staplers.

### Follow-up and recurrence

The follow-up period was defined as the time between the date of the operation and the date of the last telephone interview. Recurrence was defined as a recurrent episode of spontaneous pneumothorax after thoracoscopic surgery on chest radiography requiring invasive treatment.

All patients had at least 1 year of follow-up to be included in the study. A telephone interview was conducted to evaluate their present symptoms and postoperative recurrence of pneumothorax.

### Statistical analysis

Categorical variables and continuous variables were presented as percentages and medians ± standard deviation, respectively. Categorical variables of the two groups of propensity score-matched patients were compared by chi-square test or the Fisher’s exact test and continuous variables were compared by independent-samples t-test. The multivariate logistic regression was used to calculate the propensity score. Statistical analysis was performed by using SPSS software (version 23.0, SPSS Inc., Chicago, Illinois, USA). A *p* < 0.05 with two-sided was considered statistically significant.

## Results

### Patient characteristics

Of the 127 patients who met inclusion criteria, 32 were treated by subxipoid approach, 95 by transthoracic approach. The baseline characteristics of 127 patients are summarized in Table 1.

**Table 1:** Patients characteristics before and after propensity score matching

Characteristics	Unmatched group			Matched group		
	Transthoracic	Subxiphoid	*p*-value	Transthoracic	Subxiphoid	*p*-value
Age (years)	26.43 ± 11.57	21.56 ± 4.38	0.001	20.47 ± 5.80	21.56 ± 4.38	0.398
Gender			0.015			1.0
Male	73	31		31	31	
Female	22	1		1	1	
Height (kg)	58.39 ± 8.36	61.09 ± 9.25	0.126	59.42 ± 8.29	61.09 ± 9.25	0.449
Weight (cm)	172.68 ± 7.49	177.13 ± 5.56	0.003	177.09 ± 4.29	177.13 ± 5.56	0.980
Body mass index (BMI)	19.61 ± 2.79	19.41 ± 2.32	0.718	18.86 ± 2.56	19.41 ± 2.32	0.377
Side involved			0.238			0.895
Left	48	12		13	12	
Right	36	13		14	13	
Bilateral	11	7		5	7	

### Propensity score matching estimation

Patients in both groups were matched 1-to-1 with respect to clinical variables. The matching based on propensity scores produced 32 patients in each group. After propensity score matching, age, gender, BMI, and side involved were well balanced between the paired groups. The *P* values were recalculated and there were no significant differences between the two groups.

In terms of operative factors, showed in Table 2, patients who underwent transthoracic approach had less operation time (*p* = 0.004). There were no differences between the groups with respect to the hospital stay after surgery, chest drain duration, and the number of the staples used for the operation. The subgroup analysis showed that the operation time for bilateral bullectomy was similar between the groups (*p* = 0.986). Postoperative recurrence occurred in 1/32 of patients in both group without any significant differences. The arrhythmic events which occurred during surgery is more frequently in subxiphoid approach than transthoracic approach (*p* = 0.011). There was no conversion to open thoracotomy in both groups. Regarding laboratory data, white blood cell counts on postoperative day 1 (119,100 vs. 140,900/ll) decreased in transthoracic approach compared with subxiphoid approach (*p* = 0.018). There were one cases of incision infection in subxiphoid approach group.There were no cases of postoperative bleeding, or mortality in either group.

**Table 2:** Postoperative events after propensity score matching

	Transthoracic	Subxiphoid	*p*-value
Operative time (min)	57.31 ± 34.95	80.47 ± 27.04	0.004
No. of staples	2.66 ± 1.41	2.53 ± 1.67	0.747
Drainage (day)	5.22 ± 3.23	5.53 ± 3.17	0.698
Postoperative stay (days)	8.34 ± 3.70	10.13 ± 4.57	0.091
Postoperative recurrence	0	1	1.0
Arrhythmia during sugery	0	7	0.011

## Discussion

Transthoracic thoracoscopic surgery is the most commonly employed approach to the thoracic cavity. Liu CC [4] reported that they had performed a case of thoracoscopic lobectomy through the subxiphoid approach as an access to the chest for a patient with lung cancer in the left upper lobe. Recently, Hernandez-Arenas reported their initial experience of 153 consecutive patients with uniportal, video-assisted, subxiphoid approach to major lung resection [7].

However, to the best of our knowledge, the uniportal subxiphoid thoracoscopic surgery is performed only in a limited number of hospitals due to its technical difficulty. In the present study, the safety and effectiveness of transthoracic thoracoscopic surgery and uniportal subxiphoid thoracoscopic surgery for spontaneous pneumothorax were compared.

The results of this study revealed that postoperative recurrence, the hospital stay after surgery, chest drain duration, and the number of the staples used for the operation were similar. The operation time was longer in subxiphoid approach group than transthoracic approach group, mostly owing to performing the dissection to the pleural space via the subxiphoid incision and the exposure of surical field. However, the operation time for bilateral bullectomy was similar between the groups. These may be due to the ability of performing the bilateral surgery through the single subxiphoid incision. The count of white blood cell on postoperative day 1 was less in transthoracic approach group than subxiphoid approach group. These results may be due to more operation time.

The arrhythmic events which occurred during surgery are more frequently in subxiphoid approach, 10/30 and 0/30, respectively. This indicates that during left sided procedures, compression of the instruments on the pericardium had resulted in transient arrhythmias in the learning curve period. As experienced in this procedure and a sternal lifter applied, this problem could be overcome.

As for the results of postoperative recurrence, there was no significantly difference between the both groups.

Since the subxiphoid method required longer operating time and more cardiac complications in this study, causions should be taked. Up to now, there is still not enough evidence to support the superiority of the subxiphoid approach to transthoracic approach for spontaneous pneumothorax, as a result of the subxiphoid approach is a relatively new procedure. However, more and more thoracic surgeons accept to perform subxiphoid approach and more and more studies on subxiphoid approach are being reported.

The present study may have potential limitations. First of all, this was a retrospective study, and no postoperative pain assessment was performed. After matching, there was one woman for 31 men, and the gender distribution varied greatly. Second, although both procedures were performed during the entire period of the study, transthoracic approach was more commonly performed. Third, there may have been differences in the accumulated experience of the surgeon, which might have resulted in seletion bias between the both groups.

## Conclusion

Although transthoracic thoracoscopic surgery for spontaneous pneumothorax is well established, as advancements in uniportal subxiphoid thoracoscopic surgery instruments and the accumulation of further surgical experiences, uniportal subxiphoid thoracoscopic surgery may be a potentially alternative way to management of patients with spontaneous pneumothorax in selected cases, especially for bilateral surgery.

## Abbreviation

BMI: body mass index
